# Analysis of factors associated with severe disease and construction of a monogram for elderly patients with community-acquired pneumonia complicated by heart failure

**DOI:** 10.12669/pjms.42.8.14924

**Published:** 2026-08

**Authors:** Yanli Sun, Simiao Yu, Qianli Zhan, Jianlong Li

**Affiliations:** 1 Yanli Sun, General Practice Three Department, Baoding NO.1 Central Hospital, Baoding 071000, Hebei, China; 2 Simiao Yu, General Practice Three Department, Baoding NO.1 Central Hospital, Baoding 071000, Hebei, China; 3 Qianli Zhan, General Practice Three Department, Baoding NO.1 Central Hospital, Baoding 071000, Hebei, China; 4 Jianlong Li Department of Cardiology, Affiliated Hospital of Hebei University, Baoding 071000, Hebei, China

**Keywords:** Community-acquired pneumonia, Disease severity, Elderly, Heart failure, Nomogram, Risk prediction

## Abstract

**Objective:**

To identify independent influencing factors of severe disease among old community-acquired pneumonia(CAP) patients complicated by heart failure(HF), and to establish the nomogram model for identifying severe illness early in clinical settings.

**Methodology:**

This was a retrospective study. Altogether 140 elderly patients with concurrent CAP and HF admitted into Baoding No.1 Central Hospital between January 2022 to November 2025. According to the CURB-65 (confusion, blood urea nitrogen, respiratory rate, systolic blood pressure, age ≥65 years) score, patients were classified as the non-high-risk(*n =*100) or high-risk(*n =*40) group. Later, demographic and baseline data, infection and inflammatory markers, cardiac and other organ function parameters, coagulation parameters, systemic parameters, and clinical manifestations were compared between these two groups.

**Results:**

After preliminary screening through univariate analysis, multivariate logistic regression identified procalcitonin(PCT), B-type natriuretic peptide(BNP), and impaired consciousness as independent predictors of severe disease(*P<* 0.05, respectively). These significant predictors were incorporated into a nomogram model to estimate individual risk. The ROC curve analysis showed that the combined model yielded an area under the curve of 0.891(95% confidence interval: 0.818–0.963). Moreover, our calibration curve closely aligned with the ideal curve, indicating that our predicted risk was highly consistent with the actual risk. As for clinical utility, the DCA revealed substantial net clinical benefits of our constructed model among a broad threshold probability spectrum(0.05–1.0).

**Conclusion:**

The nomogram model incorporating PCT, BNP, and impaired consciousness demonstrates greater predictive power than those using any single parameter alone. With good calibration and favorable clinical utility.

## INTRODUCTION

Community-acquired pneumonia(CAP) is the lung parenchymal infectious inflammatory process that occurs outside hospital setting, involving pneumonia resulting from pathogens with a known incubation period that manifest clinically only after admission. CAP serves as the major infectious factor inducing mortality, which has become an increasingly severe health issue as population aging deepens. This imposes a heavy burden on healthcare systems worldwide. Given the age-related decline in physiological reserve, diminished immunity, and slower recovery, the elderly population is more susceptible to pneumonia and related complications.^1^

In clinical practice, CURB-65 score has been extensively adopted to assess CAP severity. This scoring system comprises five items, namely, confusion, blood urea nitrogen >7.0 mmol/L, respiratory rate (RR) ≥30 breaths/min, systolic blood pressure <90 mmHg or diastolic blood pressure ≤60 mmHg, alongside age ≥65 years. Heart failure (HF) is defined as a broad syndrome caused by abnormal cardiac structure and/or function that influences ventricular ejection and/or filling. Clinically, patients with HF often present with fatigue, dyspnea, and signs of fluid retention, such as pulmonary congestion, systemic circulation congestion, and peripheral edema.^2^ The current work reviewed clinical data from 140 elderly patients with concurrent CAP and HF, aiming to identify factors that independently contribute to severe disease. On this basis, we further constructed and validated a nomogram model that facilitated to timely and accurately identify high-risk cases, so as to provide clinicians with a quantitative tool to support risk stratification and timely intervention.

## METHODOLOGY

We retrospectively analyzed 140 elderly patients with CAP complicated by HF who were admitted into the Baoding No.1 Central Hospital between January 2022 to November 2025. According to the modified CURB criteria issued by the British Thoracic Society^3^, these patients were assigned to the non-high-risk (NHR) (*n =*100) or high-risk (HR)(*n =*40) group.

### Ethical approval:

The study was approved by the Institutional Ethics Committee of Baoding No.1 Central Hospital (No.:[2025]262; approval; Date: November 10, 2025), and written informed consent was obtained from all participants.

### Inclusion criteria:

- Confirmed diagnosis of CAP and HF.
- Age ≥65 years.
- Measurement of neutrophil (NE), lymphocyte (L), interleukin-6(IL-6), B-type natriuretic peptide (BNP), C-reactive protein(CRP), platelet(PLT), procalcitonin(PCT), and D-dimer(D-D) contents after admission.
- No use of antimicrobial, anticoagulant, or immunosuppressive medications before hospitalization.
- Those providing informed consent.

### Exclusion criteria:

- Pulmonary embolism, immune dysfunction, malignancy, or severe psychiatric disorders.
- Antimicrobial or immunosuppressive therapy within one week before admission.
- Other infectious diseases, such as cardiopulmonary resuscitation, trauma, postoperation, burn, shock, sunstroke, neuroendocrine neoplasm, extracorporeal circulation, liver cirrhosis, pancreatitis, mesenteric necrosis and catheter infections.
- Incomplete clinical data.

Electronic medical records were retrieved to collect clinical data, including demographic and baseline characteristics, inflammatory markers, infection markers, cardiac and other organ function parameters, coagulation parameters, systemic parameters, and clinical manifestations. The NHR group contained 58 males whereas 42 female patients, and their average age and average disease duration were 76.9 ± 9.28 years and of 9.48 ± 3.00 days separately. Additionally, the HR group consisted of 25 males whereas 15 female patients, and their average age and average disease duration were 78.5 ± 6.76 years and 10.3 ± 2.42 days separately. Sex composition, age, comorbidities (including hypertension, diabetes, cerebral infarction, and chronic obstructive pulmonary disease), the New York Heart Association (NYHA) functional classification of heart failure, IL-6, white blood cell count(WBC), NE, left ventricular ejection fraction(LVEF), left ventricular end-diastolic diameter(LVEDD), partial pressure of oxygen in arterial blood(PaO_2_), D-D, fibrinogen(FIB), RR, fever, cough, and dyspnea were not significantly different between two groups (all *P>* 0.05).

After admission, all patients received standard foundational treatments for both CAP and HF. Management included antimicrobial therapy, expectorants, and, depending on cardiac function, diuretics, vasodilators, or other supportive measures. Before initiating therapy, peripheral venous blood was collected for measurement of NE, L, BNP, IL-6, CRP, PCT, PLT, and D-D levels. The neutrophil-to-lymphocyte ratio(NLR) was calculated as NE divided by L. NE, L, CRP, and PLT were measured using the Sysmex XN1000 analyzer. D-D was assessed with the Sysmex CS5100. PCT and IL-6 were determined using a Thermo Fisher time-resolved fluorescence immunoassay system. BNP levels were measured via fluorescence immunoassay. All blood samples were processed within thirty minutes of collection. Calibration was performed before sample testing, and coefficients of variation remained within acceptable limits to ensure analytic reliability. The findings of the echocardiogram at admission were recorded simultaneously.

### Outcome measures:

- The two groups were compared for demographic and baseline characteristics, inflammatory markers, infection markers, cardiac and other organ function parameters, coagulation parameters, systemic parameters, and clinical manifestations.
- Univariate alongside multivariate logistic regression was performed for identifying independent risk factors related to severe disease among elderly CAP patients complicated by HF.
- The nomogram prediction model was also constructed by incorporating independent risk factors, whereas receiver operating characteristic (ROC) curves, calibration curves, as well as decision curve analysis (DCA) were utilized for assessing model discrimination, accuracy and clinical usefulness separately.

### Statistical analysis:

SPSS 26.0 was employed for statistical analysis. Results were performed normality and homogeneity of variance tests. Normally-distributed data are expressed as mean ± standard deviation (*x̄*±*s*). Comparisons of means among multiple groups were performed using one-way analysis of variance, and pairwise comparisons were conducted with independent-samples t-tests. Non-normally distributed data are presented as medians with interquartile ranges, and analyzed by rank-sum tests. Chi-square (χ²) test was employed to analyze categorical data. Univariate alongside multivariate logistic regression was carried out for identifying independent risk factors. The identified independent predictors were incorporated for constructing the nomogram model. Model discrimination was assessed by area under the curve (AUC). Calibration curves were plotted to compare predicted and observed probabilities. Net clinical benefit was assessed by DCA. A *P*<0.05 indicating a statistical significance.

## RESULTS

Sex composition, age, comorbidities (like hypertension, diabetes, cerebral infarction, chronic obstructive pulmonary disease), NYHA classification, IL-6, WBC, NE, LVEDD, LVEF, PaO_2_, D-D, FIB, RR, fever, cough, and dyspnea were not significantly different in both groups (all *P >* 0.05). In contrast, the two groups differed significantly in CRP, PCT, L, NLR, BNP, PLT, and the presence of impaired consciousness (all *P<* 0.05). Table-I.

**Table-I:** Clinical data in two groups.

Category	Parameter	NHR group (n =100)	HR group (n =40)	Statistic	P-value
Demographic and baseline characteristics	Sex			0.090	0.765
	Male	58(58%)	25(62.5%)		
	Female	42(42%)	15(37.5%)		
	Age (years)	76(70.75,83)	78(73,83.25)	-1.075	0.283
	Concurrent hypertension	69(69%)	25(62.5%)	0.292	0.589
	Concurrent cerebral infarction	20(20%)	4(10%)	1.369	0.242
	Concurrent diabetes	41(41%)	19(47.5%)	0.263	0.608
	Concurrent COPD	25(25%)	14(35%)	0.968	0.325
	NYHA classification			0.808	0.668
	2	4(4%)	3(7.5%)		
	3	44(44%)	16(40%)		
	4	52(52%)	21(52.5%)		
Infection and inflammatory markers	CRP	22.55(8.15,53.17)	75(41.94,138.54)	-4.705	<0.001
	PCT	0.11(0.06,0.33)	0.71(0.33,1.47)	-5.318	<0.001
	IL-6	25.1(9.12,49.4)	41.28(14.52,78.73)	-1.937	0.053
	WBC	8.67(6.23,11.27)	9.57(8.2,12.5)	-1.702	0.089
	NE	6.28(4.63,8.91)	8.1(5.62,9.86)	-1.884	0.060
	L	0.99(0.67,1.47)	0.74(0.61,0.81)	3.019	0.003
	NLR	6.52(3.73,10.07)	10.32(6.67,13.07)	-2.858	0.004
Cardiac and other organ function parameters	BNP	440.5(212.25,796.75)	859.5(655.75,1390)	-4.654	<0.001
	LVEDD	4.8(4.48,5.3)	4.6(4.38,5.43)	0.914	0.914
	LVEF	55(46,59)	51(47.75,58)	0.493	0.493
	PaO_2_	83.4(69.48,101.75)	81(60.88,88.88)	0.101	0.101
Coagulation and systemic parameters	D-D	1.04(0.58,2.01)	1.26(0.6,2.34)	-1.086	0.278
	FIB	3.58(2.82,5.24)	3.97(3.2,4.66)	-0.348	0.907
	PLT	201.5(153,258.5)	178(148.75,196)	2.286	0.022
	RR	20(19,20)	20(20,22)	-1.257	0.182
	Impaired consciousness	56(56%)	33(82.5%)	7.558	0.006
Clinical manife-stations	Fever	32(32%)	18(45%)	1.575	0.209
	Cough	85(85%)	29(72.5%)	2.183	0.140
	Dyspnea	79(79%)	26(65%)	2.287	0.130

Variables identified as potentially relevant from univariate regression were further examined with the multivariate logistic regression model. PCT, BNP, and impaired consciousness were found to be independent predictors of severe illness(all *P<* 0.05). Table-II.

**Table-II:** Multivariate analysis of factors associated with severe disease.

Variable	β	SE	OR (95% CI)	P-value
Constant	-4.960	1.704	0.007(0.000–0.198)	0.004
CRP	0.004	0.006	1.004(0.992–1.015)	0.556
PCT	0.241	0.081	1.273(1.086–1.492)	0.003
L	–0.752	0.718	0.472(0.115–1.928)	0.295
NLR	-0.031	0.032	0.970(0.911–1.032)	0.331
BNP	0.002	0.001	1.002(1.001–1.004)	0.008
PLT	-0.002	0.004	0.998(0.990–1.006)	0.636
Impaired consciousness (present vs. absent)	2.685	1.273	14.662(1.21–177.72)	0.035

**Fig.1:**
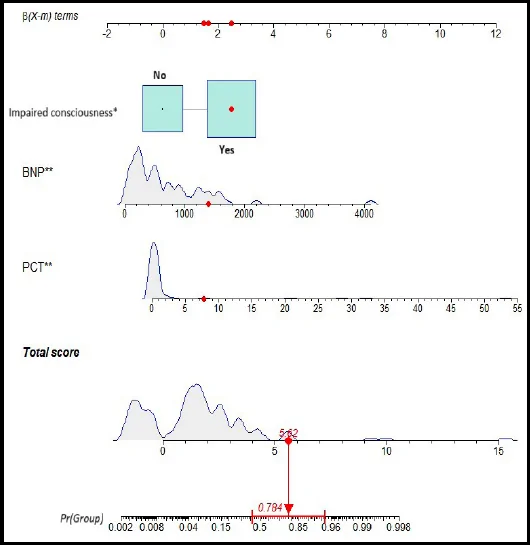
Nomogram predictive model.

PCT, BNP, and impaired consciousness were used for developing the nomogram model for estimating the severe disease risk (Fig.1). The model provides an individualized risk score by assigning weighted points to each predictor. For example, a patient presenting with impaired consciousness, a PCT level of 7.76 ng/L, and a BNP value of 1,400 pg/mL would correspond to a total point score translating to an estimated 78.4% probability of severe illness.

**Fig.2:**
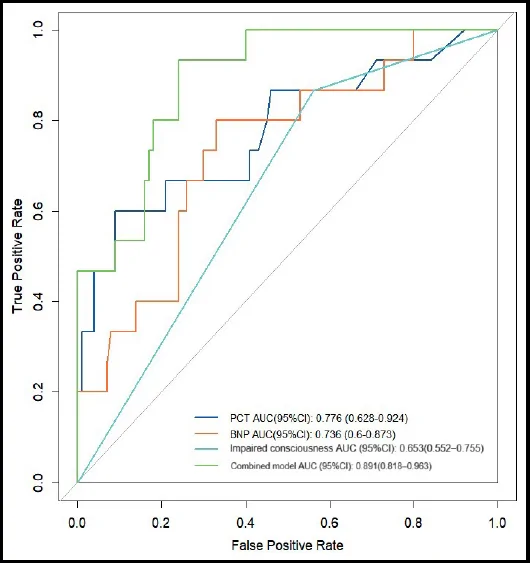
ROC curve.

The ROC curve analysis showed that the AUC of the combined model was 0.891 (95% confidence interval [95% CI]: 0.818–0.963) for predicting severe disease, outperforming those with any single predictor alone(PCT [AUC = 0.776], BNP [AUC = 0.736], or impaired consciousness [AUC = 0.653]). Fig-2. Table-III.

**Table-III:** Performance metrics of different nomogram predictive models.

Model	Sensitivity	Specificity	Accuracy	F1	AUC (95%CI)
Combined model	0.933	0.760	0.783	0.528	0.891(0.818–0.963)
PCT	0.600	0.910	0.870	0.545	0.776(0.628–0.924)
BNP	0.800	0.670	0.687	0.400	0.736(0.600–0.873)
Impaired consciousness	0.867	0.440	0.496	0.310	0.653(0.552–0.755)

**Fig.3:**
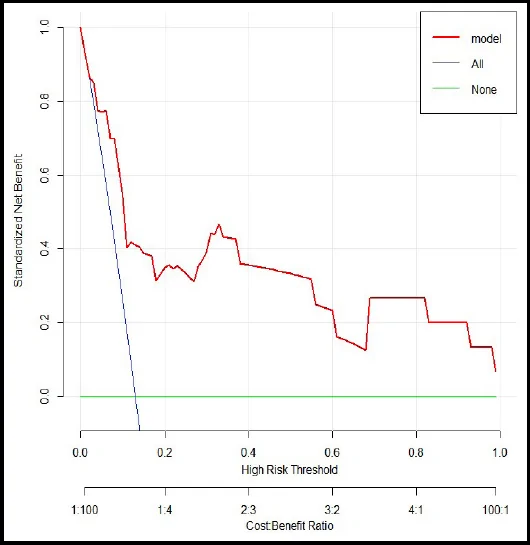
DCA of the nomogram model.

Fig.3 presents the DCA for the nomogram, where the x-axis represents the threshold probability, while the y-axis shows the net benefit. The “ALL” line reflects the extreme scenario in which every patient receives intervention; as the threshold probability increases, which implies a higher penalty for unnecessary intervention, the net benefit of treating everyone naturally decreases. Conversely, the “None” line assumes that no patient is treated, whose net benefit was zero across all thresholds. According to DCA result, our constructed model achieved the clear net clinical benefit within the threshold probability scope of 0.05-1.0, suggesting meaningful clinical utility across this interval.

**Fig.4:**
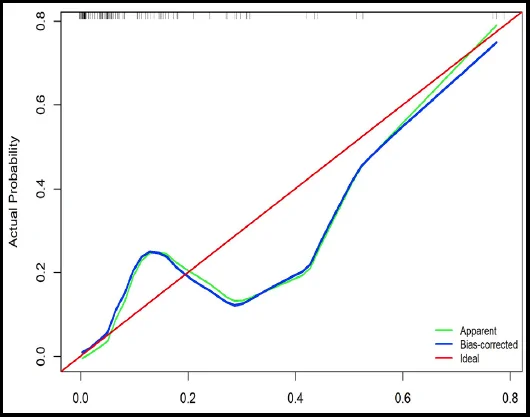
Calibration curve of the nomogram predictive model

Predictive accuracy was assessed by comparing predicted risks with observed outcomes using a calibration curve. From Fig.4, the close alignment of calibration curve with ideal line indicated strong agreement between estimated probabilities and actual incidences. The *P-*value by Hosmer-Lemeshow test was 0.3979(χ² = 8.3728), higher than 0.05, verifying no significant calibration deviation.

## DISCUSSION

We identified that PCT independently predicted the risk of severe disease in elderly patients with CAP complicated by HF (odds ratio [OR]: 1.273, 95% CI: 1.086–1.492). Differing from our findings, Wu Y et al.^4^ reported that serum PCT levels at admission did not serve as a reliable biomarker for severe disease in elderly patients with CAP complicated by HF. This may be related to the fact that our study cohort presented with more severe HF and infection, and that we adopted a more integrative analytical approach. In line with our results, Hoppe JM et al.^5^ found that pneumonia further elevated PCT levels in elderly patients with HF and worsened their clinical condition. PCT is a protein released directly in response to endotoxins or indirectly via cytokines (*e.g*., IL-6). It is widely used as a biomarker for the clinical diagnosis of bacterial infections. However, elevated PCT levels can also be found under non-infectious conditions, such as cardiogenic shock, trauma, heat shock, autoimmune disorders, acute graft-versus-host disease, and paraneoplastic syndromes. In patients with HF complicated by bacterial infection, the diagnostic threshold for PCT should be correspondingly elevated.^6^ Existing literature has suggested that in patients with HF and pneumonia, the increase in inflammatory markers may be attributed to the following mechanisms: first, pulmonary infection stimulates the secretion of inflammatory cytokines and induces immune activation, thereby increasing PCT expression in peripheral blood; second, considering the central role of bacteria among the pathogens involved in pneumonia, PCT production is jointly enhanced by bacterial endotoxins and host inflammatory cytokines.^7^-^10^

More than 70% of BNP is released from the ventricles. Hemodynamic overload induced by increased cardiac volume and/or pressure leads to elevated wall tension, while ischemia and hypoxia can upregulate BNP gene expression. As a result, BNP, with its vasodilatory, natriuretic, and diuretic effects, improves myocardial relaxation and reduces myocardial fibrosis.^6^ Reportedly, toxins produced by pneumonia pathogens can impair cardiac function, leading to HF and elevating BNP levels.^11^,^12^ In our multivariate logistic regression analysis, BNP was the independent risk factor related to severe disease among old CAP patients complicated by HF (OR: 1.002, 95% CI: 1.001–1.004). Nevertheless, BNP alone did not demonstrate strong predictive value for severe disease in our study cohort.

The mortality rate increases with age, particularly beyond 65 years.^13^ Reportedly, nearly 70% of CAP cases in older adults are aspiration pneumonia.^14^ Considering the high prevalence of dysphagia, cough reflex dysfunction, and consciousness impairment among older adults, silent aspiration is considered the major mechanism responsible for aspiration pneumonia.^15^ Previous studies have shown that renal function, D-D, CRP, blood glucose levels, hypoproteinemia, NLR, pleural effusion, bilateral involvement, and multilobar infiltration are key indicators for assessing disease severity and prognosis in elderly patients with CAP.^16^,^17^ In patients with HF, pulmonary edema compromises mucociliary clearance in the respiratory tract, leading to intrapulmonary bacterial accumulation and predisposing patients to secondary pneumonia.^18^ Pneumonia, in turn, can trigger acute decompensation of HF. The coexistence of pneumonia and HF can lead to exacerbation of patients’ condition, especially in older adults with reduced physiological reserve. In this population, treatment is particularly challenging, and the mortality rate is significantly higher.^19^ In many elderly patients with chronic HF, common clinical manifestations include cough, fatigue, weariness, poor appetite, and lethargy rather than overt dyspnea. Among older adults with CAP, altered mental status and psychiatric symptoms are often observed at the initial onset.^20^ This highlights the importance of a timely, accurate assessment of a patient’s condition for improving patient outcomes.

Notably, we also identified that impaired consciousness was the independent predictor for severe disease (OR: 14.662, 95% CI: 1.21–177.72). This highly aligns with the clinical characteristics of elderly patients, such as decompensation, susceptibility to central nervous system infection, and rapid systemic deterioration. This manuscript conform the Enhancing the quality and Transparency Of health Research (EQUATOR) network guidelines.

### Limitations:

First, the small sample size and retrospective design probably lead to unavoidable bias. In addition, this nomogram has not yet been externally validated. To overcome these limitations, prospective, large-scale multicenter studies should be conducted for validating and optimizing this model and enhancing its scientific robustness.

## CONCLUSIONS

Our study identified PCT, BNP, and impaired consciousness as independent predictors of severe disease in elderly patients with CAP complicated by HF through multivariate analysis. Using these predictors, we developed a nomogram model that demonstrated good discrimination, calibration, and clinical utility upon validation. This model provides an intuitive, efficient quantitative tool to timely identify high-risk cases, offering a valuable reference for clinical practice.

### Authors’ Contributions:

**YS and SY:** Designed this study, Manuscript writing and are responsible and accountable for the accuracy or integrity of the work.

**SY:** Literature search, Collected and analyzed clinical data. Critical review.

**QZ:** Participated in acquisition, analysis, or interpretation of data and draft the manuscript.

All authors have read and approved the final manuscript.
